# Early exercise intervention promotes myelin repair in the brains of ischemic rats by inhibiting the MEK/ERK pathway

**DOI:** 10.1515/tnsci-2022-0335

**Published:** 2024-03-14

**Authors:** Junyi Wang, Xinyu Ding, Chen Li, Chuan Huang, Changkai Ke, Chunlei Xu, Chunxiao Wan

**Affiliations:** Department of Physical Medicine and Rehabilitation, Tianjin Medical University General Hospital, No. 154 Anshan Road, Heping District, Tianjin, 300052, China

**Keywords:** middle cerebral artery occlusion, early exercise intervention, myelin

## Abstract

Our previous studies have shown that early exercise intervention after stroke increases neural activity and synaptic plasticity and promotes the recovery of nerve fiber bundle integrity in the brain. However, the effect of exercise on the repair of myelin in the brain and the related mechanism are still unclear. In this study, we randomly divided the rats into three groups. Before and after 28 days of intervention, body weight, nerve function, the infarct size, white matter fiber bundle integrity, and nerve myelin structure and function were observed by measuring body weight, analysis of modified neurological severity score, CatWalk gait analysis, MRI, luxol fast blue staining, immunofluorescence, and transmission electron microscopy. Changes in the expression of proteins in the MEK/ERK pathway were assessed. The results showed that early exercise intervention resulted in neurological recovery, decreased the infarct volume and increased nerve fiber integrity, the myelin coverage area, myelin basic protein (MBP) fluorescence intensity expression, and myelin thickness. Furthermore, the expression level of MBP was significantly increased after early exercise intervention, while the expression levels of p-MEK1/2 and p-ERK1/2 were significantly reduced. In the cell study, MBP expression levels were significantly higher in the oxygen and glucose deprivation and administration group.In summary, early exercise intervention after stroke can promote myelin repair by inhibiting the MEK/ERK signaling pathway.

## 1 Introduction

Stroke is the second leading cause of death and the third leading cause of death and disability worldwide today [1]. Stroke is one of the leading causes of death in China, and its incidence is continuing to rise [2]. Stroke is usually classified as either ischemic or hemorrhagic, and 87% of stroke cases are ischemic [3]. Rehabilitation can promote functional recovery in stroke patients [4]. Early rehabilitation exercise after stroke to promote the recovery of neurological function has attracted increasing attention. In the early stage of stroke, exercise intervention can increase sensation, movement, strength, endurance, and function in patients [5]. Existing studies have found that the recovery of motor function after stroke can be mediated by neuroplasticity [6]. After stroke, neuroplasticity refers to a large amount of activity-dependent recombination of brain structure, which promotes partial functional recovery, reflected not only by changes in neurons and synapses but also in myelin repair, which also plays an important role in the normal function and restoration of neural networks [7]. Studies have found that exercise training can increase postsynaptic excitability and neuronal activity in layer V pyramidal neurons in the motor cortex of mice and increase the myelination of axons [8].

Myelin plays roles in neural transmission, nutritional support, and protection [9] and is vulnerable to damage in neurological diseases [10]. Oligodendrocytes (OLs) are injured or killed and white matter fibers are injured after stroke, which is one of the common causes of neurological dysfunction in adults. In recent years, many studies have confirmed that myelin plasticity may be the basis of myelin repair after injury in humans and animals, especially youth [11,12]. An increasing number of studies have shown that promoting myelin repair can improve prognosis by restoring neuronal transmission and promoting axonal survival [13]. Studies have shown that some specifically timed interventions can promote oligodendrogenesis and the involvement of OLs in myelin repair, which may enhance myelin repair and accelerate the recovery of patients with demyelinating diseases [14]. However, enhancing myelin repair and reducing repair failure is still the greatest challenge in the field of stroke [15].

Studies have found that the MEK/ERK signaling pathway can regulate myelin plasticity in models of experimental autoimmune encephalomyelitis and demyelinating diseases [16]. In Parkinson’s disease models, exercise intervention can inhibit the overactivation of the MEK/ERK pathway and alleviate motor dysfunction [17]. Besides, early MEK1/2 inhibition improves long-term functional outcome, and promotes recovery processes after stroke [18]. However, whether early exercise after cerebral infarction can inhibit MEK/ERK signaling and promote myelin repair and remodeling is not well studied.

In this study, we observed changes in body weight, modified neurological severity score (mNSS), the infarct volume, white matter fiber integrity, myelin morphology, and the expression of MEK/ERK signaling pathway components in middle cerebral artery occlusion (MCAO) rats before and after early exercise intervention. In addition, MO3.13 cells were subjected to oxygen and glucose deprivation (OGD) and treated with PD0325901 (PD, a MEK inhibitor), and changes in MEK, ERK, and myelin basic protein (MBP) expression in the cells after 4 days of treatment were assessed to explore the influence of exercise intervention on the myelin sheath after cerebral ischemia and the role of the MEK/ERK signaling pathway in this effect.

## 2 Materials and methods

### 2.1 Experimental animals and groups

Adult male Sprague‒Dawley rats (8–10 weeks old, weighing 280–320 g, Beijing Huafukang Biotechnology Co., Ltd, China) were used in this study. They were randomly divided into three groups: the sham operation (sham) group, MCAO with sedentary intervention (MCAO-SED) group, and MCAO with exercise intervention (MCAO-EX) group. Early exercise intervention was initiated 1 day after confirming the successful establishment of the MCAO model. The animals were killed 4 weeks later, and follow-up tests were carried out. The experimental procedure was performed according to the guidelines of the National Research Council’s Guide for the Care and Use of Laboratory Animals and approved by the Laboratory Animal Welfare Ethics Committee of Tianjin Medical University General Hospital (IRB2021-DWFL-403), and the number of animals used and the pain of animals during the experiment were minimized. All tests were double-blind.

### 2.2 MCAO model

The modified Longa thread embolization method was used to construct the MCAO model [19]. Under anesthesia (pentobarbital sodium, 40 mg/kg, ip), the rats were fixed in the supine position, and a median incision was made along the carotid artery. All tissue layers were bluntly separated, the left common carotid artery and the internal and external carotid artery were separated, and a medical nylon monofilament with fine silicon coating (2838-A4, 0.38 ± 0.02 mm, Beijing Xinnong Technology Co., LTD, China) was inserted into the left internal carotid artery to block the middle cerebral artery (approximately 18.0–20.0 mm from the distal end of the carotid artery) for 60 min. Then, the monofilament was removed to induce reperfusion. Once the rats recovered, Longa scores (1–3) were assigned to determine the success of modeling.

### 2.3 Exercise intervention program

A previously described exercise program was used in this study [20]. All rats were randomly divided into different groups, and exercise intervention began 1 day after MCAO. The rats were allowed to exercise on a treadmill (ZS-PT, Beijing, China, an angle of 0° and a speed of 12 m/min) for 30 min daily five times a week for 4 weeks.

### 2.4 Body weight measurement and neurological function assessment

Changes in the body weight and neurological function of rats were assessed at 1, 3, 7, 14, 21, and 28 days after MCAO. mNSS, ranging from 0 to 18, were used to assess sensory function, motor function, balance, and reflexes (Table A1). The more severe the neurological deficits were, the higher the score.

### 2.5 Gait analysis

On the 28th day after MCAO, the CatWalk system was used to test the rats in each group. Each rat was evaluated at least three times. Each time, a set length glass plate was crossed in a specified time (within 10 s), and the entire experimental process was completed in a dark and silent environment. The gait parameters were automatically calculated by the analysis software (CatWalk XT 10.6).

### 2.6 MRI scan

On Days 1, 14, and 28 of the exercise intervention, the rats were anesthetized (inhaled 3% isoflurane) and underwent magnetic resonance imaging (3T, MR750, General Electric, USA) to collect T2-weighted images (T2WI, FOV = 6 × 6 mm, matrix = 192 × 192 mm, TR = 1,000 ms, TE = 70 ms, thickness = 2 mm, and number of slices = 18), and the infarct location was determined. The infarct volume ratio was calculated using the following equation: infarct volume ratio = (total volume of the contralateral hemisphere − noninfarcted volume of the ipsilateral hemisphere)/total volume of the contralateral hemisphere. Myelin integrity was measured by determining the fractional anisotropy (FA) value and apparent diffusion coefficient (ADC) of the injured internal capsule and the corresponding contralateral internal capsule by diffusion tensor imaging (DTI). Relative FA (rFA) = the FA value of the affected side/the FA value of the unaffected side. Relative ADC (rADC) = the ADC of the affected side/the ADC of the unaffected side.

### 2.7 Luxol fast blue (LFB) staining

Brain tissue was embedded in paraffin, and then 5 μm thick coronal sections were immersed in 0.1% LFB (LFB, Sigma, S3382) at 60°C for 2 h. The slices were soaked in a 0.05% lithium carbonate solution to differentiate white matter from gray matter. Finally, the sections were placed in distilled water and stained with cresyl violet solution at room temperature for 30–40 s before washing with distilled water. Then, myelin staining was observed under an optical microscope.

### 2.8 Immunofluorescence

Brain tissues were immersed in 4% paraformaldehyde for 12 h before being completely dehydrated in 15 and 30% sucrose solutions, rinsed with PBS, and blotted dry. Brain tissue sections were prepared at a thickness of 10 μm on a −20°C cryostat (Leica CM1860, Germany). The frozen sections were rewarmed for 30 min, washed three times with PBS, and blocked with 3% bovine serum albumin (Solarbio, China) for 1 h. Then, an anti-MBP primary antibody (1:500; Bioss, China) diluted with antibody diluent (Solarbio, China) was added to the slices overnight at 4°C. The slices were then incubated with Alexa Fluor 488-conjugated goat anti-rabbit secondary antibody (1:200; Invitrogen, USA) at room temperature for 1 h. 4,6-diamidino-2-phenyl indole (Abcam, UK) was added to stain the nuclei, and the slices were sealed. Images of the area around the infarction were taken using an inverted fluorescence microscope (Olympus IX73, Japan). Six myelin structures were randomly selected in each field of view, and the fluorescence intensity was analyzed using ImageJ software.

### 2.9 Transmission electron microscopy (TEM)

Brain tissue was cut into 1 mm thick sections using a rat coronal section mold, and sections containing the area of the external capsule (1 mm^3^) surrounding the infarction were soaked in 2.5% glutaraldehyde at a low temperature for 24 h, incubated in 0.1 M phosphate buffer (PB) three times, and fixed with 1% osmium tetroxide for 2 h. The samples were soaked in 0.1 M PB three times, dehydrated, embedded, cut into slices of 50–70 nm, placed on a copper cell grid, and stained with uranyl acetate and lead citrate. Myelin morphology and thickness were measured using a transmission electron microscope (Hitachi HT7700, Japan).

### 2.10 Cell culture

MO3.13 human OL cells were cultured in six-well plates and divided into three groups, namely, the control group, OGD group, and OGD supplemented with MEK inhibitor (OGD + PD) group. Cells in the control group were left untreated. In the OGD group, the cells were washed twice with PBS, the medium was replaced with glucose-free Dulbecco's modified eagle medium (DMEM), and the cells were incubated in 95% N_2_/5% CO_2_; Subsequently, the cells were incubated in 94% N_2_/5% CO_2_/1% O_2_ for 4 h, and then the medium was replaced with high-glucose DMEM containing 4 μg/mL dimethyl sulfoxide (DMSO). Cells in the OGD + PD group were treated in the same way described above, but PD0325901 (10 μM) was added. Treatment lasted for 4 days [16].

### 2.11 Western blot

Total proteins were extracted from peri-infarct brain tissue and MO3.13 cells for western blotting. The total protein concentration was determined using a BCA kit (Solarbio, China). The proteins were separated by polyacrylamide gel electrophoresis (SDS‒PAGE) and transferred to a polyvinylidene fluoride membrane. The membrane was incubated with 5% skim milk at room temperature for 1 h and treated with anti-MBP (1:2,000; PTM Biolab, China), anti-MEK/2 (1:500; Affinity, China), anti-ERK1/2 (1:1,000; Affinity, China), anti-p-ERK1/2 (1:500; Affinity, China), anti-p-MEK1/2 (1:500; Affinity, China), and anti-GAPDH (1:1,000; PTM Biolab, China) antibodies at 4°C overnight. The membrane was then incubated with either anti-rabbit IgG (1:8,000; CST, USA) or anti-rat IgG (1:8,000; CST, USA) at room temperature for 1 h. Enhanced chemiluminescence solution (Millipore, Germany) was used to visualize the protein bands.

### 2.12 Statistical analysis

SPSS 25.0 (SPSS Inc., Armonk, NY, USA) and GraphPad Prism 9.0 (GraphPad Software, Inc., La Jolla, CA, USA) were used for statistical analysis. The data are expressed as the mean ± standard error (SEM). The Shapiro‒Wilks test was performed to verify the normal distribution of the data. Multiple groups were compared using univariate analysis of variance (ANOVA), the independent sample Kruskal‒Wallis test and the LSD-t *post hoc* test. *P* < 0.05 was considered to indicate statistical significance.


**Ethical approval:** The research related to animals use has been complied with all the relevant national regulations and institutional policies for the care and use of animals. The animal study was reviewed and approved by Laboratory Animal Welfare Ethics Committee of Tianjin Medical University General Hospital (IRB2021-DWFL-403). The MO3.13 cell line was provided by BeNa Culture Collection, Ltd.

## 3 Results

### 3.1 Preparation and verification of the MCAO model

**Figure 1:**
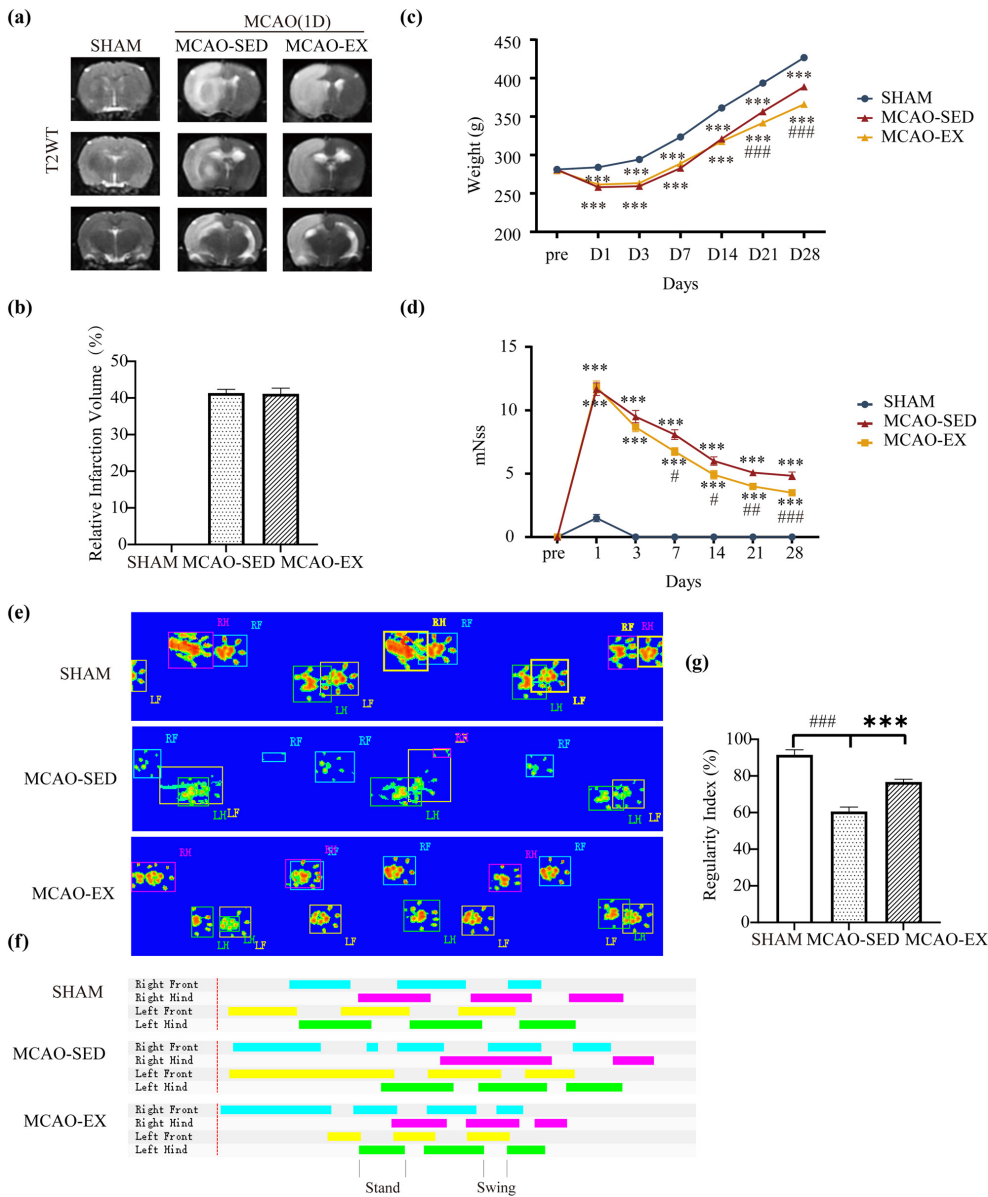
MNSS scores and status of the MCAO rats. (a) and (b) Representative T2WI images of each group and cerebral infarction volume ratio of rats in each group at 24 h after MCAO. *N* = 6/group. (c) Changes in the body weight of rats 28 days after MCAO. *N* = 12/group. (d) Changes in the mNSS scores of rats 28 days after MCAO. *N* = 12/group. (e) and (f) Representative paw step images and limbs’ supporting timing view of CatWalk gait analysis. (g) Quantitative analysis of catwalk at Day 28. *N* = 6/group. Compared with the sham group: *P < 0.05, **P < 0.01, and ***P < 0.001; compared with the MCAO-SED group: #*P* < 0.05, ##*P* < 0.01, and ###*P* < 0.001. Mean ± SEM.

MRI was performed 24 h after MCAO. The results showed that there was no significant difference in the cerebral infarct volume ratio between the MCAO-SED and MCAO-EX groups before early exercise intervention (*P* = 0.808, Figure 1a and b).

### 3.2 Effects of a 28-day exercise intervention on the state of rats with cerebral infarction

The body weight of the rats in the three groups increased with time (Figure 1c), and the body weights of the MCAO-SED group and MCAO-EX group were lower than that of the sham group (*P* < 0.05). On Day 21, the body weight of the MCAO-EX group was lower than that of the MCAO-SED group (*P* < 0.001). On the 28th day, the body weight of the MCAO-EX group was significantly lower than that of the MCAO-SED group (*P* < 0.001).

### 3.3 Effect of a 28-day exercise intervention on neurological function in rats with cerebral infarction

Intragroup comparisons showed that, compared with Day 1, the mNSS of both the MCAO-SED and MCAO-EX groups decreased significantly on Day 3 (*P* < 0.001) (Figure 1d).

Intergroup comparisons showed that compared with the MCAO-SED group, the mNSS of the MCAO-EX group decreased significantly on Day 7 (*P* < 0.05), and this trend continued until the end of intervention on Day 28.

Comparisons of regularity index of rats in different groups showed that on the 28th day post stroke, the regularity of gait was significantly decreased in the MCAO-SED group (*P* < 0.001), and it was significantly improved by exercise (*P* < 0.001, Figure 1e–g).

### 3.4 Effect of a 28-day exercise intervention on infarct volume in rats with cerebral infarction

**Figure 2:**
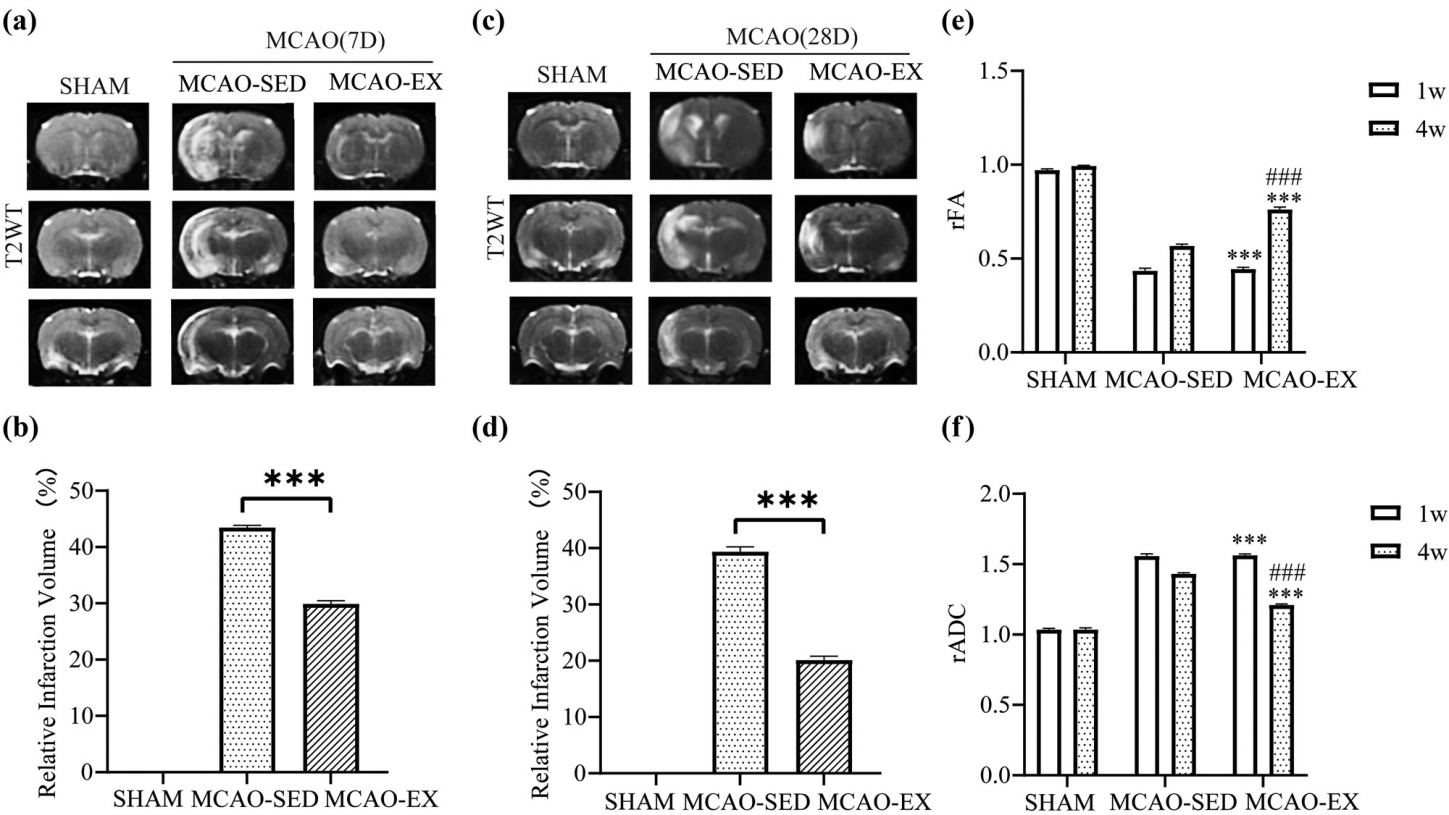
MRI images of rats. (a) and (b) Representative T2WI images of each group and cerebral infarction volume ratio of rats in each group on the 7th day of intervention. (c) and (d) Representative T2WI images of each group and cerebral infarction volume ratio of rats in each group on the 28th day of intervention. (e) rFA values after intervention. (f) rADC values after intervention. *P < 0.05, **P < 0.01, and ***P < 0.001: the MCAO-EX group compared with the MCAO-SED group. #*P* < 0.05, ##*P* < 0.01, and ###*P* < 0.001: self-comparison of the MCAO-SED group and the MCAO-EX group on Day 28. ANOVA. Mean ± SEM. *N* = 6/group.

MRI was performed after 7 and 28 days of intervention (Figure 2a–d), and the results showed that after intervention, the infarct volume ratio in the MCAO-EX group was significantly reduced compared to that in the MCAO-SED group (*P*_7d_ < 0.001, *P*_28d_ < 0.001). The infarct volume ratio in the MCAO-EX group was significantly lower on Day 28 than on Day 7 (*P* < 0.001). The infarct volume ratio in both groups decreased over time (*P*_SED_ < 0.001, *P*_EX_ < 0.001). Statistical analysis showed that the effects of time and intervention method on the infarct volume ratio were statistically significant [*F*_time (1,15)_ = 99.829, *P*_time_ < 0.001; *F*_intervention (2,15)_ = 4411.179, *P*_intervention_ < 0.001], and there was an interaction between time and intervention method [*F*_time × intervention (2,15)_ = 37.555, *P* < 0.001].

### 3.5 Effects of a 28-day exercise intervention on nerve fibers in rats with cerebral infarction

After 7 and 28 days of intervention, the rFA value in the MCAO-EX group was significantly lower than that in the MCAO-SED group (*P*_7d_ < 0.001, *P*_28d_ < 0.001; Figure 2e). Intragroup comparisons showed that the rFA value in the MCAO-EX group was significantly lower at 28 days than at 7 days (*P* < 0.001). Statistical analysis showed that the effects of time and intervention method on the rFA value were statistically significant [*F*_time (1,15)_ = 774.119, *P*_time_ < 0.001; *F*_intervention (2,15)_ = 1145.054, *P*_intervention_ < 0.001], and there was an interaction between time and intervention method [*F*_time × intervention (2,15)_ = 229.793, *P* < 0.001].

After intervention, the infarct volume ratio in the MCAO-EX group was significantly reduced compared to that in the MCAO-SED group (*P*_7d_ < 0.001, *P*_28d_ < 0.001; Figure 2f). On Day 28 of intervention, the rADC value of the MCAO-EX group was significantly decreased compared to that on Day 7 (*P* < 0.001). Statistical analysis showed that the effects of time and intervention mode on the rADC value were statistically significant [*F*_time (1,15)_ = 303.856, *P*_time_ < 0.001; *F*_intervention (2,15)_ = 1354.333, *P*_intervention_ < 0.001], and there was an interaction between time and intervention method [*F*_time × intervention (2,15)_ = 128.281, *P* < 0.001].

According to the MRI results, exercise intervention can improve the integrity of fiber tracts after MCAO.

### 3.6 Effect of a 28-day exercise intervention on myelin sheaths in rats with cerebral infarction

**Figure 3:**
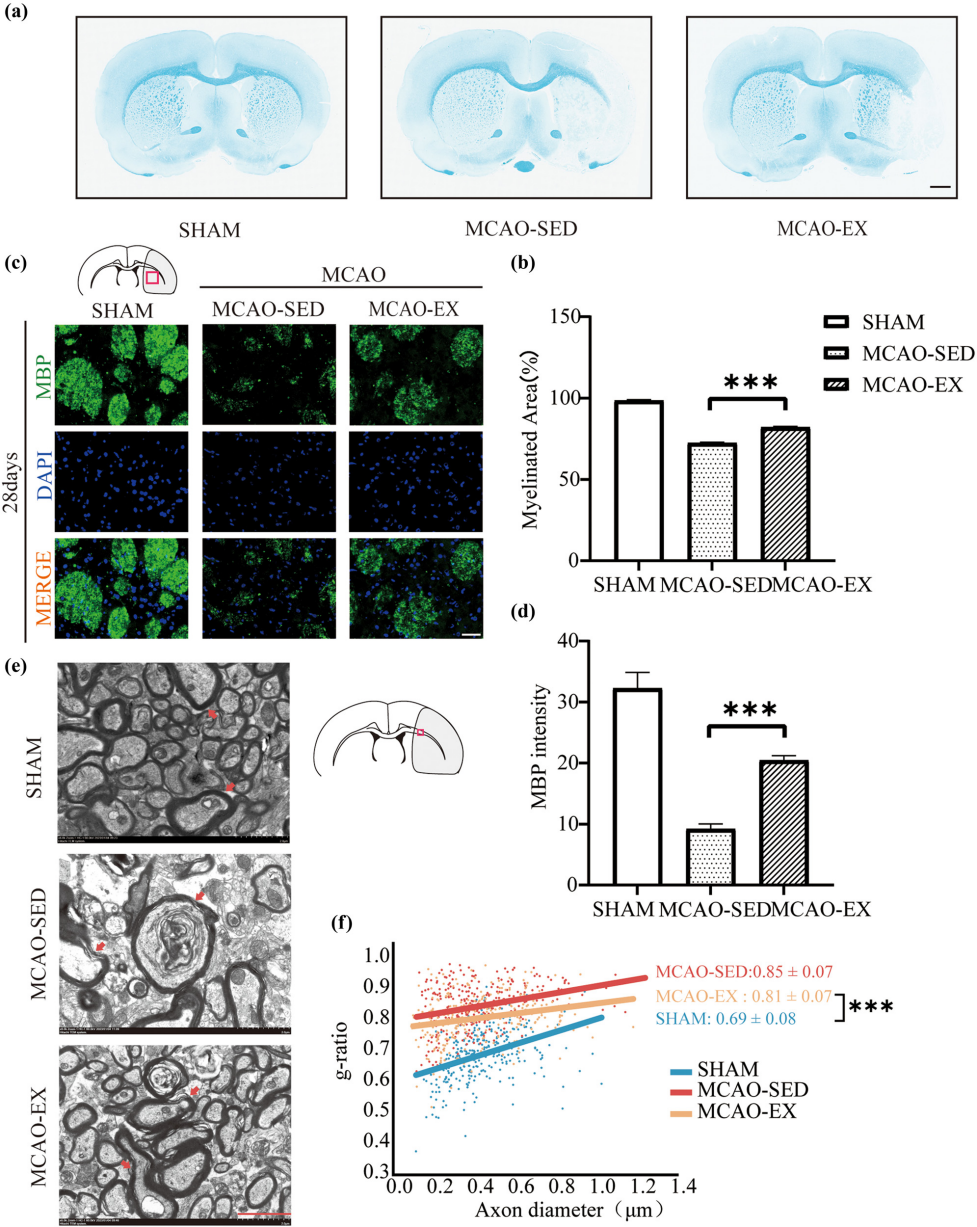
Myelin integrity assessment. (a) and (b) LFB staining of the ischemic penumbra striatum 28 days after MCAO. Scale bar = 1,000 μm. (c) and (d) Immunohistochemical staining of MBP and quantification of MBP expression in rats 28 days after MCAO (red rectangle in the figure). Scale bar = 100 μm. One-way ANOVA and the LSD-t *post hoc* test. Mean ± SEM. *N* = 6/group. (e) and (f) TEM analysis of myelin integrity in the external capsule (EC) on Day 28 after MCAO. Scale bar = 2 μm. *P < 0.05, **P < 0.01, and ***P < 0.001: the MCAO-EX group compared with the MCAO-SED group. Independent sample Kruskal‒Wallis test. Mean ± SEM. *N* = 300/group. MBP, myelin basic protein.

**Figure 4:**
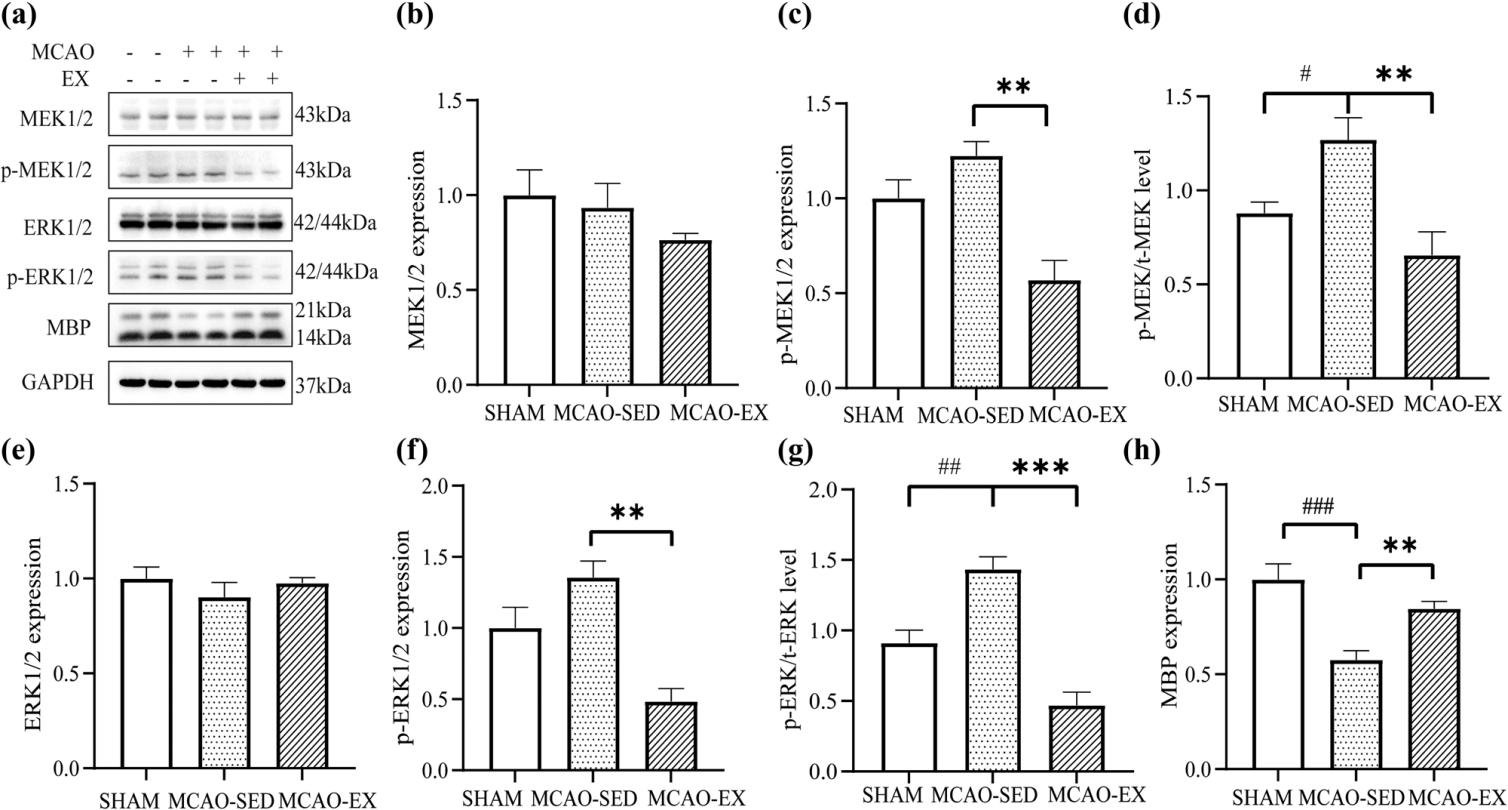
Protein expression in the ischemic penumbra. (a)–(h) At 28 days after MCAO, the expression levels of MEK1/2, p-MEK1/2, ERK1/2, p-ERK1/2, and MBP in penumbra tissues were analyzed by western blotting. #*P* < 0.05, ##*P* < 0.01, and ###*P* < 0.001: the MCAO-SED group compared with the sham group. *P < 0.05, **P < 0.01, and ***P < 0.001: the MCAO-EX group compared with the MCAO-SED group. One-way ANOVA and the LSD-t *post hoc* test. Mean ± SEM. The experiment was repeated three times.

After 28 days of intervention, myelin fibers in brain sections from the sham group were stained by LFB, the whole brain was uniformly stained, wrapped myelin fibers were dense and uniform, and myelin fibers were intact (Figure 3a). The integrity of myelin was decreased in the brain tissue sections from the MCAO-SED and MCAO-EX groups, and some areas were not stained with LFB. The degree of myelin loss was decreased and myelin coverage was increased in the MCAO-EX group compared with the MCAO-SED group (*P* < 0.001). Immunofluorescence (Figure 3c) showed that the expression of MBP in the ischemic striatum of the MCAO-SED group was significantly lower than that of the sham group 28 days after intervention (*P* < 0.001). The expression of MBP in the MCAO-EX group was significantly increased compared to that in the MCAO-SED group (*P* < 0.001). The ultrastructure of myelin was observed using TEM (Figure 3e). The ratio of the inner axon diameter to the total outer diameter of myelin fibers (g-ratio) was used to assess myelin thickness. The results showed that in the MCAO-SED group, the myelin sheaths in the demyelinated area of the external capsule were thinner, the damaged myelin layer was fractured and expanded, the myelin sheaths around axons were discontinuous, axons were loosely wrapped by abnormal myelin sheaths, and the g-ratio was decreased (*P* < 0.001). The thickness of the myelin sheath in the demyelinated area was increased and the myelin sheath surrounding axons was denser in the MCAO-EX group, and the g-ratio in the MCAO-EX group was increased compared to that in the MCAO-SED group (*P* < 0.001). MBP staining showed that the expression level of MBP in the MCAO-EX group was significantly higher than that in the MCAO-SED group (*P* < 0.01) (Figure 4f).

### 3.7 Effect of a 28-day exercise intervention on the expression of MEK/ERK signaling pathway components in rats with cerebral infarction

We measured the total protein expression and phosphorylation of ERK1/2 and its upstream molecule MEK1/2 in the ischemic penumbra (Figure 4a–e). The results showed that compared with the sham group, the total expression of MEK1/2 and ERK1/2 in the ischemic penumbra were unchanged in the MCAO-SED group (*P*_MEK_ = 0.674, *P*_ERK_ = 0.258). There was no difference in total MEK1/2 and ERK1/2 levels in the ischemic penumbra between the MCAO-EX and MCAO-SED groups (*P*_MEK_ = 0.282, *P*_ERK_ = 0.387). Compared with the sham group, the levels of p-MEK and p-ERK in the MCAO-SED group were increased. Compared with the MCAO-SED group, the levels of p-MEK1/2 (*P* < 0.01) and p-ERK1/2 (*P* < 0.01) in the MCAO-EX group were significantly decreased. However, the ratio of p-MEK/t-MEK and p-ERK/t-ERK was significantly higher in MCAO-SED group compared with the sham group (*P* < 0.05, *P* < 0.01), and significantly lower in MCAO-EX group compared with the MCAO-SED group (*P* < 0.01, *P* < 0.001).

### 3.8 Effect of MEK/ERK pathway inhibition on myelin repair by OLs after OGD in cell experiments

**Figure 5:**
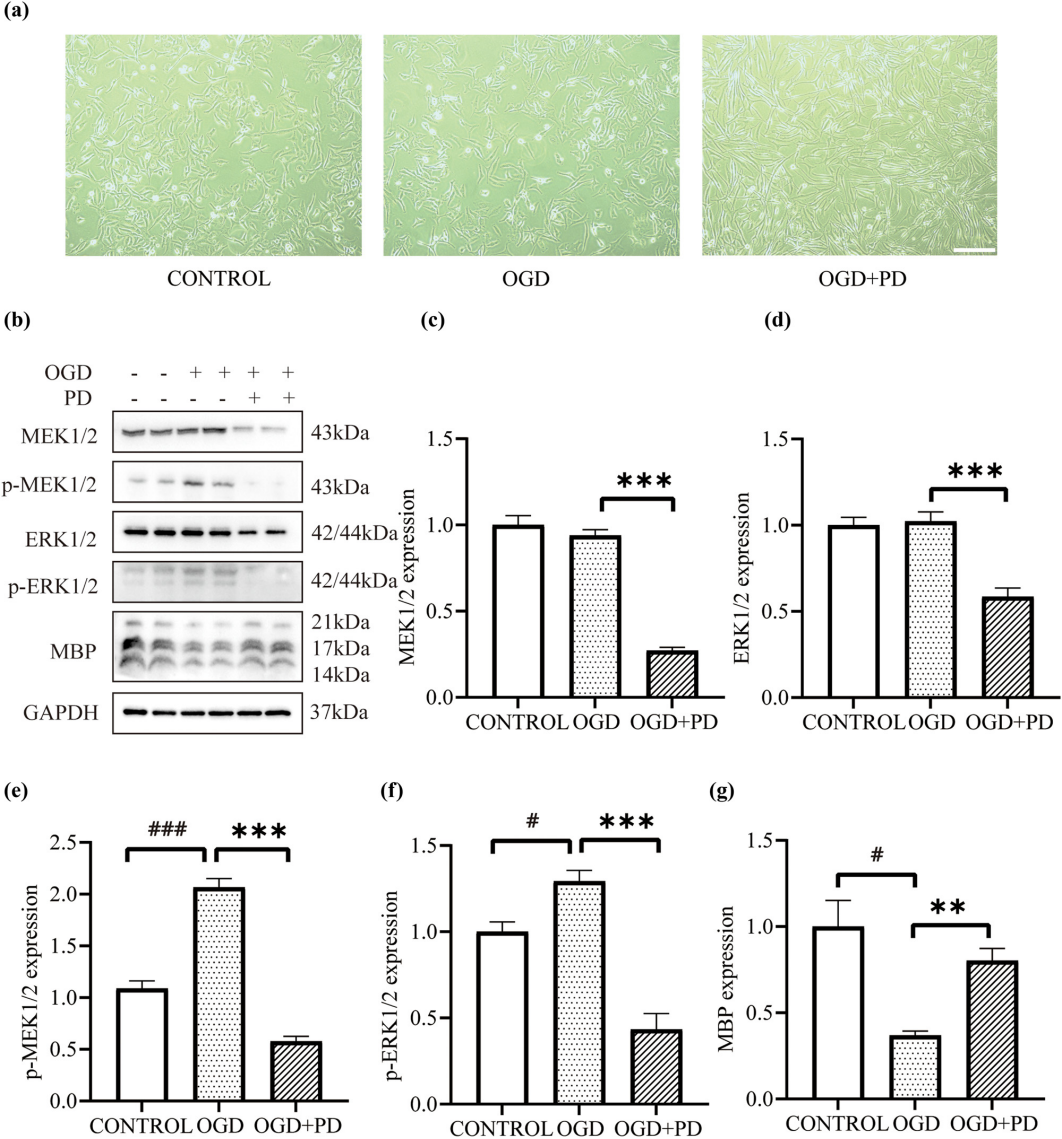
Analysis of morphology and protein expression. (a) Cells from each group were observed under an optical microscope after 4 h of OGD and 4 days of reperfusion. Scale bar = 1,000 μm. (b–g) The expression of MEK1/2, p-MEK1/2, ERK1/2, p-ERK1/2, and MBP in MO3.13 cells was analyzed by western blotting after 4 h of OGD and 4 days of reperfusion. #*P* < 0.05, ##*P* < 0.01, and ###*P* < 0.001: the OGD group compared with the control group. *P < 0.05, **P < 0.01, and ***P < 0.001: the OGD + PD group compared with the OGD group. One-way ANOVA and the LSD-t *post hoc* test. Mean ± SEM. The experiment was repeated three times.

In the cell experiment, MO3.13 OLs were subjected to OGD for 4 h and treated with DMSO or the MEK inhibitor PD0325901 for 4 days during reperfusion, and cell morphology was observed by optical microscopy. The results showed that MO3.13 cells in the OGD group had few short cell processes and exhibited reduced refraction, mild swelling, and partial retraction. The cell processes in the OGD + PD group were long and clear, and the refraction did not change significantly (Figure 5a). The total expression and phosphorylation of MEK1/2 and ERK1/2 were determined by extracting proteins from each group. The results showed (Figure 5b–e) that compared with those in the OGD group, the total expression and phosphorylation of MEK1/2 and ERK1/2 were significantly decreased in the OGD + PD group (*P*_MEK_ < 0.001, *P*_p-MEK_ < 0.001, *P*_ERK_ < 0.001, *P*_p-ERK_ < 0.001). In addition, the levels of p-MEK and p-ERK in the OGD group were significantly higher than those in the control group (*P*_p-MEK_ < 0.001, *P*_p-ERK_ < 0.05). Analysis of MBP expression showed (Figure 5f) that MBP expression in the OGD + PD group was significantly higher than that in the OGD group (*P* < 0.01).

## 4 Discussion

### 4.1 Early exercise intervention improved body weight and behavior in MCAO rats

Studies have indicated that exercise intervention, which is a crucial neurological rehabilitation method after stroke, promotes functional recovery, walking and balance after stroke [21]. After 7 days of grip training immediately after cerebral ischemia, gripping ability was found to be nearly restored to the level observed before the injury [22]. Our team’s previous studies have shown that early exercise intervention after cerebral infarction can reduce the infarct volume and improve nerve function [23]. In this study, both behavior and body weight were changed in the MCAO-EX group compared with the MCAO-SED group. In the intervention conducted 21 days piror, there was no significant difference in weight between the MCAO-SED and MCAO-EX groups. However, after 21 days, the weight of the MCAO-EX group was significantly lower than that of the MCAO-SED group, which may have been because exercise increased the number of calorie burned, leading to weight loss; there was no further observed change in body composition between the two groups in this study. The mNSS of both the MCAO-SED and MCAO-EX groups decreased significantly on Day 3, suggesting spontaneous restoration of nerve function after stroke. On the 7th day of intervention, the mNSS of the MCAO-EX group was significantly lower than that in the MCAO-SED group, suggesting that early exercise can promote the recovery of neurological function in animals with cerebral infarction. In addition, the results of gait analysis showed that the regularity index of the MCAO-EX group was greatly improved. The recovery of nerve function depends on the repair of nerves and reinnervation, including restoration of synaptic structure and function, the enhancement of interhemispheric connections, the promotion of nerve regeneration, the acceleration of nerve functional reorganization, and the promotion of compensation outside the infarct tissue [24]. Recent studies have found a strong correlation between increased functional connectivity and improved motor performance of limbs [25]. However, insufficient myelination in the brains of mice may lead to neurological dysfunction after stroke [26]. The repair of myelin after stroke is associated with motor and functional impairment [27], which further indicates the importance of myelin repair for the recovery of motor function after stroke.

### 4.2 Exercise intervention improved fiber bundle integrity and reduced the infarct volume in MCAO rats

Before intervention, there was no significant difference in the cerebral infarct volume ratio between the MCAO-SED group and the MCAO-EX group. MRI showed that the 28-day exercise intervention reduced the cerebral infarct volume. DTI showed that the rFA and rADC values of the MCAO-EX group were significantly different from those of the MCAO-SED group. This indicates the restoration of the integrity of nerve fiber bundles [28]. DTI can not only reflect the integrity of fiber bundles through noninvasive methods but also has high sensitivity for detecting myelination [29]. The changes in rFA and rADC values reflect an increase in myelin repair.

### 4.3 Exercise intervention promoted myelin repair in cerebral infarction rats

Myelin plays an important role in increasing conduction speed and providing metabolic support [30]. Its structure is delicate and complex, and it is susceptible to damage caused by multiple factors. Animal studies have shown that cerebral ischemia can lead to loss of OLs, leading to demyelination and impaired axon function [26]. Long-term axonal integrity and neuronal survival are maintained by OLs [31], and loss of the normal fine structure of the myelin sheath can lead to delayed axonal degeneration and even premature death [32]. Therefore, myelin repair after stroke is also very important.

In this study, TEM and histology were used to observe the structural features of the myelin sheath after stroke. We found that after stroke, the myelin sheath was thinner and covered a smaller area. The results of protein and immuonfluorescence showed that the expression of MBP was decreased after stroke. These changes were significantly ameliorated after exercise intervention, indicating that exercise promotes myelin repair.

### 4.4 Exercise promoted myelin repair by inhibiting the MEK/ERK pathway

Previous studies have demonstrated that early exercise intervention not only promotes neurogenesis and myelin repair in pups after stroke [33] but also reduces the phosphorylation of the ERK and JNK proteins [34,35]. Our results showed that phosphorylated MEK and ERK levels in the MCAO-EX group was significantly reduced compared with those in the MCAO-SED group on Day 28, which was consistent with the results of previous studies. However, there was no significant difference in the total protein expression of MEK and ERK in the MCAO-EX group compared with the MCAO-SED group, indicating direct inhibition of MEK and ERK phosphorylation by exercise.

Studies have shown that inhibiting MEK/ERK signal activation may be a promising therapeutic approach for the treatment of myelin injury after stroke [16]. In this study, the expression of MBP protein was significantly increased after the application of MEK inhibitors *in vitro*, which also confirmed that inhibiting the MEK/ERK pathway can promote myelin repair.

The role of ERK1/2 signaling in central nervous system OLs is complex and controversial. Studies have shown that abnormal low-density lipoprotein receptor expression significantly inhibits the Shc/MEK/ERK pathway in chronic cerebral ischemia (CCH) models, leading to OL death after CCH [36]. Another study showed that extracellular vesicles derived from Wharton’s jelly mesenchymal stromal cells promoted the maturation of MO3.13 OLs by reducing MEK/ERK signaling [37]. The reason why our results differ from those of the previous study may be because the cells used in the previous study were MO3.13 cells, which differ from the primary OL line used in the current study. Due to the complex and multistep process of myelination, inhibition of ERK1/2 signaling can inhibit the maturation of early progenitor cells to late progenitor cells, leading to a subsequent reduction in the number of immature OLs [38], which may be the reason for the difference in the conclusion of this study from that of different studies.

Previous studies on cerebral ischemia have focused more on neuronal recovery. Exercise can promote the recovery of neurons after stroke, but can it also promote the recovery of glial cells? What mechanism plays the role still needs to be further explored. MEK/ERK is a common signaling pathway, but whether it is affected by stroke or exercise intervention is unknown. In this article, the common path as an attempt to verify, first verify the results of the trend, in order to inspire other ideas. It is hoped that the future can be combined with drug use and other aspects to play a multiplier effect, and lay the foundation for future in-depth research.

## 5 Conclusion

Previous studies by our team have shown that early exercise intervention can promote synaptic growth in the ischemic penumbra in adult rats and regulate neuroplasticity [20,23]. This study focused on the mechanism of myelin repair after ischemic stroke in rats. The results showed that the myelin sheath in the penumbra was damaged after cerebral ischemia, and exercise intervention promoted myelin repair and rehabilitation after ischemic stroke in rats. In addition, it was shown that the MEK/ERK signaling pathway is involved in myelin repair stimulated by exercise intervention.

## Appendix

**Table A1:** mNSS

**Motor tests**	6
**Raising mouse by tail**	3
Flexion of forelimb	1
Flexion of hindlimb	1
Head moved >10° to vertical axis within 30 s	1
**Placing mouse on ϐloor (normal = 0; maximum = 3)**	3
Normal walk	0
Inability to walk straight	1
Circling toward paretic side	2
Falls down to paretic side	3
**Sensory tests**	8
**Placing test (visual and tactile test)**	1
**Proprioceptive test (deep sensation, pushing paw against table edge to stimulate limb muscles)**	1
**Beam balance tests (normal = 0; maximum = 6)**	6
Balances with steady posture	0
Grasps side of beam	1
Hugs beam and 1 limb falls down from beam	2
Hugs beam and 2 limb fall down from beam, or spins on beam (>60 s)	3
Attempts to balance on beam but falls off (>40 s)	4
Attempts to balance on beam but falls off (>20 s)	5
Falls off; no attempt to balance or hang on to beam (<20 s)	6
**Reflex absence and abnormal movements**	4
Pinna reflex (head shake when auditory meatus is touched)	1
Corneal reflex (eye blink when cornea is lightly touched with cotton)	1
Startle reflex (motor response to a brief noise from snapping a clipboard paper)	1
Seizures, myoclonus, myodystonia	1
**Maximum points**	**18**

## References

[1] Valery LF, Benjamin AS, Catherine OJ, Cao L, Gregory AR, Catherine B, et al. Global, regional, and national burden of stroke and its risk factors, 1990–2019: a systematic analysis for the Global Burden of Disease Study 2019. Lancet Neurol. 2021;20(10):795–820. 10.1016/S1474-4422(21)00252-0 PMC8443449 34487721

[2] Tu WJ, Zhao Z, Yin P, Cao L, Zeng J, Chen H, et al. Estimated burden of stroke in China in 2020. JAMA Netw Open. 2023;6(3):e231455. 10.1001/jamanetworkopen.2023.1455 PMC9982699 36862407

[3] Ajoolabady A, Wang S, Kroemer G, Penninger JM, Uversky VN, Pratico D, et al. Targeting autophagy in ischemic stroke: from molecular mechanisms to clinical therapeutics. Pharmacol Ther. 2021;225:107848. 10.1016/j.pharmthera.2021.107848 PMC8263472 33823204

[4] Zhang T, Zhao J, Li X, Bai Y, Wang B, Qu Y, et al. Chinese Stroke Association guidelines for clinical management of cerebrovascular disorders: executive summary and 2019 update of clinical management of stroke rehabilitation. Stroke Vasc Neurol. 2020;5(3):250–9. 10.1136/svn-2019-000321 PMC7548515 32595138

[5] Han P, Zhang W, Kang L, Ma Y, Fu L, Jia L, et al. Clinical evidence of exercise benefits for stroke. Adv Exp Med Biol. 2017;1000:131–51. 10.1007/978-981-10-4304-8_9 29098620

[6] Mang CS, Campbell KL, Ross CJD, Boyd LA. Promoting neuroplasticity for motor rehabilitation after stroke: considering the effects of aerobic exercise and genetic variation on brain-derived neurotrophic factor. Phys Ther. 2013;93(12):1707–16. 10.2522/ptj.20130053 PMC3870490 23907078

[7] Jia W, Kamen Y, Pivonkova H, Káradóttir RT. Neuronal activity-dependent myelin repair after stroke. Neurosci Lett. 2019;703:139–44. 10.1016/j.neulet.2019.03.005 30904575

[8] Chen K, Zheng Y, Wei J-A, Ouyang H, Huang X, Zhang F, et al. Exercise training improves motor skill learning via selective activation of mTOR. Sci Adv. 2019;5(7):eaaw1888. 10.1126/sciadv.aaw1888 PMC6609215 31281888

[9] Cristobal CD, Lee HK. Development of myelinating glia: an overview. Glia. 2022;70(12):2237–59. 10.1002/glia.24238 PMC9561084 35785432

[10] Liu H, Yang Y, Xia Y, Zhu W, Leak RK, Wei Z, et al. Aging of cerebral white matter. Ageing Res Rev. 2017;34:64–76. 10.1016/j.arr.2016.11.006 PMC5250573 27865980

[11] de Faria O, Pivonkova H, Varga B, Timmler S, Evans KA, Káradóttir RT. Periods of synchronized myelin changes shape brain function and plasticity. Nat Neurosci. 2021;24(11):1508–21. 10.1038/s41593-021-00917-2 34711959

[12] Almeida RG, Lyons DA. On myelinated axon plasticity and neuronal circuit formation and function. J Neurosci. 2017;37(42):10023–34. 10.1523/JNEUROSCI.3185-16.2017 PMC6596541 29046438

[13] Caprariello AV, Adams DJ. The landscape of targets and lead molecules for remyelination. Nat Chem Biol. 2022;18(9):925–33. 10.1038/s41589-022-01115-2 PMC9773298 35995862

[14] Bacmeister CM, Barr HJ, McClain CR, Thornton MA, Nettles D, Welle CG, et al. Motor learning promotes remyelination via new and surviving oligodendrocytes. Nat Neurosci. 2020;23(7):819–31. 10.1038/s41593-020-0637-3 PMC7329620 32424285

[15] Chen D, Huang Y, Shi Z, Li J, Zhang Y, Wang K, et al. Demyelinating processes in aging and stroke in the central nervous system and the prospect of treatment strategy. CNS Neurosci Ther. 2020;26(12):1219–29. 10.1111/cns.13497 PMC7702227 33210839

[16] Suo N, Guo Y-E, He B, Gu H, Xie X. Inhibition of MAPK/ERK pathway promotes oligodendrocytes generation and recovery of demyelinating diseases. Glia. 2019;67(7):1320–32. 10.1002/glia.23606 PMC6593996 30815939

[17] Wang X, Wang Y, Chen J, Li J, Liu Y, Chen W. Aerobic exercise improves motor function and striatal MSNs-Erk/MAPK signaling in mice with 6-OHDA-induced Parkinson’s disease. Exp Brain Res. 2022;240(6):1713–25. 10.1007/s00221-022-06360-4 PMC8985567 35384454

[18] Mostajeran M, Wetterling F, Blixt FW, Edvinsson L, Ansar S. Acute mitogen-activated protein kinase 1/2 inhibition improves functional recovery and vascular changes after ischaemic stroke in rat-monitored by 9.4 T magnetic resonance imaging. Acta Physiol (Oxford, Engl). 2018;223(1):e12985. 10.1111/apha.12985 29055086

[19] Longa EZ, Weinstein PR, Carlson S, Cummins R. Reversible middle cerebral artery occlusion without craniectomy in rats. Stroke. 1989;20(1):84–91. 10.1161/01.str.20.1.84 2643202

[20] Li C, Hu J, Liu W, Ke C, Huang C, Bai Y, et al. Exercise intervention modulates synaptic plasticity by inhibiting excessive microglial activation via exosomes. Front Cell Neurosci. 2022;16:953640. 10.3389/fncel.2022.953640 PMC9345504 35928570

[21] Ali A, Tabassum D, Baig SS, Moyle B, Redgrave J, Nichols S, et al. Effect of exercise interventions on health-related quality of life after stroke and transient ischemic attack: a systematic review and meta-analysis. Stroke. 2021;52(7):2445–55. 10.1161/STROKEAHA.120.032979 34039033

[22] Zhang R, Chopp M, Zhang ZG. Oligodendrogenesis after cerebral ischemia. Front Cell Neurosci. 2013;7:201. 10.3389/fncel.2013.00201 PMC3810592 24194700

[23] Li C, Ke C, Su Y, Wan C. Exercise intervention promotes the growth of synapses and regulates neuroplasticity in rats with ischemic stroke through exosomes. Front Neurol. 2021;12:752595. 10.3389/fneur.2021.752595 PMC8581302 34777222

[24] Xing Y, Bai Y. A review of exercise-induced neuroplasticity in ischemic stroke: pathology and mechanisms. Mol Neurobiol. 2020;57(10):4218–31. 10.1007/s12035-020-02021-1 32691303

[25] Lu Y, Lin Z, Li M, Zhuang Y, Nie B, Lei J, et al. Three-phase enriched environment improves post-stroke gait dysfunction via facilitating neuronal plasticity in the bilateral sensorimotor cortex: a multimodal MRI/PET analysis in rats. Neurosci Bull. 2023;(10). 10.1007/s12264-023-01155-1 PMC11178725 38055107

[26] Li L, Li R, Zacharek A, Wang F, Landschoot-Ward J, Chopp M, et al. ABCA1/ApoE/HDL signaling pathway facilitates myelination and oligodendrogenesis after stroke. Int J Mol Sci. 2020;21(12):4369–87. 10.3390/ijms21124369 PMC7352241 32575457

[27] Lakhani B, Hayward KS, Boyd LA. Hemispheric asymmetry in myelin after stroke is related to motor impairment and function. NeuroImage Clin. 2017;14:344–53. 10.1016/j.nicl.2017.01.009 PMC5312556 28229041

[28] Pinter D, Gattringer T, Fandler-Höfler S, Kneihsl M, Eppinger S, Deutschmann H, et al. Early progressive changes in white matter integrity are associated with stroke recovery. Transl Stroke Res. 2020;11(6):1264–72. 10.1007/s12975-020-00797-x PMC7575507 32130685

[29] Cerina M, Muthuraman M, Gallus M, Koirala N, Dik A, Wachsmuth L, et al. Myelination- and immune-mediated MR-based brain network correlates. J Neuroinflammation. 2020;17(1):186. 10.1186/s12974-020-01827-z PMC7293122 32532336

[30] Salzer JL, Zalc B. Myelination. Curr Biol. 2016;26(20):R971–R5. 10.1016/j.cub.2016.07.074 27780071

[31] Saab AS, Nave K-A. Myelin dynamics: protecting and shaping neuronal functions. Curr Opin Neurobiol. 2017;47:104–12. 10.1016/j.conb.2017.09.013 29065345

[32] Simons M, Nave K-A. Oligodendrocytes: myelination and axonal support. Cold Spring Harb Perspect Biol. 2015;8(1):a020479. 10.1101/cshperspect.a020479 PMC4691794 26101081

[33] Cheng J, Shen W, Jin L, Pan J, Zhou Y, Pan G, et al. Treadmill exercise promotes neurogenesis and myelin repair via upregulating Wnt/β‑catenin signaling pathways in the juvenile brain following focal cerebral ischemia/reperfusion. Int J Mol Med. 2020;45(5):1447–63. 10.3892/ijmm.2020.4515 PMC7138282 32323740

[34] Cao W, Lin J, Xiang W, Liu J, Wang B, Liao W, et al. Physical exercise-induced astrocytic neuroprotection and cognitive improvement through primary cilia and mitogen-activated protein kinases pathway in rats with chronic cerebral hypoperfusion. Front Aging Neurosci. 2022;14:866336. 10.3389/fnagi.2022.866336 PMC9198634 35721009

[35] Kim S-H, Ko YJ, Kim J-Y, Sim Y-J. Treadmill running improves spatial learning memory through inactivation of nuclear factor kappa B/mitogen-activated protein kinase signaling pathway in amyloid-β-induced Alzheimer disease rats. Int Neurourol J. 2021;25(Suppl 1):S35–43. 10.5213/inj.2142164.082 PMC8171239 34053209

[36] Xie Y, Zhang X, Xu P, Zhao N, Zhao Y, Li Y, et al. Aberrant oligodendroglial LDL receptor orchestrates demyelination in chronic cerebral ischemia. J Clin Invest. 2021;131(1):e128114. 10.1172/JCI128114 PMC7773390 33141760

[37] Joerger-Messerli MS, Thomi G, Haesler V, Keller I, Renz P, Surbek DV, et al. Human Wharton’s jelly mesenchymal stromal cell-derived small extracellular vesicles drive oligodendroglial maturation by restraining MAPK/ERK and notch signaling pathways. Front Cell Dev Biol. 2021;9:622539. 10.3389/fcell.2021.622539 PMC8044995 33869172

[38] Guardiola-Diaz HM, Ishii A, Bansal R. Erk1/2 MAPK and mTOR signaling sequentially regulates progression through distinct stages of oligodendrocyte differentiation. Glia. 2012;60(3):476–86. 10.1002/glia.22281 PMC3265651 22144101

